# Advancing Graphene Synthesis: Low-Temperature Growth and Hydrogenation Mechanisms Using Plasma-Enhanced Chemical Vapor Deposition

**DOI:** 10.3390/molecules30010033

**Published:** 2024-12-25

**Authors:** Šarūnas Meškinis, Algirdas Lazauskas, Šarūnas Jankauskas, Asta Guobienė, Rimantas Gudaitis

**Affiliations:** Institute of Materials Science, Kaunas University of Technology, K. Baršausko 59, LT-51423 Kaunas, Lithuania; sarunas.jankauskas@ktu.lt (Š.J.); asta.guobiene@ktu.lt (A.G.); rimantas.gudaitis@ktu.lt (R.G.)

**Keywords:** PECVD, graphene synthesis, low-temperature growth, hydrogenated graphene

## Abstract

This study explores the low-temperature synthesis of graphene using plasma-enhanced chemical vapor deposition (PECVD), emphasizing the optimization of process parameters to achieve controlled growth of pristine and hydrogenated graphene. Graphene films were synthesized at temperatures ranging from 700 °C to as low as 400 °C by varying methane (25–100 sccm) and hydrogen (25–100 sccm) gas flow rates under 10–20 mBar pressures. Raman spectroscopy revealed structural transitions: pristine graphene grown at 700 °C exhibited strong 2D peaks with an I(2D)/I(G) ratio > 2, while hydrogenated graphene synthesized at 500 °C showed increased defect density with an I(D)/I(G) ratio of ~1.5 and reduced I(2D)/I(G) (~0.8). At 400 °C, the material transitioned to a highly hydrogenated amorphous carbon film, confirmed by photoluminescence (PL) in the Raman spectra. Atomic force microscopy (AFM) showed pristine graphene with a root mean square roughness (*R_q_*) of 0.37 nm. By carefully adjusting PECVD synthesis parameters, it was possible to tune the surface roughness of hydrogenated graphene to levels close to that of pristine graphene or to achieve even smoother surfaces. Conductive AFM measurements revealed that hydrogenation could enhance graphene’s contact current under specific conditions. The findings highlight the role of PECVD parameters in tailoring graphene’s structural, morphological, and electronic properties for diverse applications. This work demonstrates a scalable, low-temperature approach to graphene synthesis, offering the potential for energy storage, sensing, and electronic devices requiring customized material properties.

## 1. Introduction

Graphene, known for its exceptional electrical, optical, and mechanical properties, is being developed for various optoelectronic applications, ranging from solar cells to high-speed electronics [1,2]. The giant charge carrier mobility and its multiplication, optical transparency, flexibility, very high Young’s modulus and failure stress, high ductility, and chemical inertness all account for the potential of emerging devices [3,4,5,6]. Although graphene is known as a planar sheet of carbon atoms arranged in a honeycomb-like pattern, it can present with many configurations that differ in structure and properties. Graphene flakes [7], vertical graphene [8], hydrogenated graphene films [9], and many more variants can be fabricated using chemical vapor deposition (CVD) [10].

For planar, in regard to high-quality graphene fabrication, graphite exfoliation is a method of choice, although it is not suitable for industrial applications because of the need for more scalability [11]. For large-area graphene production, the most promising technique is CVD, which is usually performed on catalytic metal (Cu, Ni, and Co) foils with subsequent transfer to the target substrate [12]. However, the downside of this is the complicated transfer process that involves careful handling of the graphene film, which can result in possible graphene contamination by different adsorbents [13], wrinkles, or ripple formation in the graphene [14]. Direct graphene growth on dielectric and semiconducting substrates has emerged as an effective approach to overcoming these limitations. One such technique, plasma-enhanced chemical vapor deposition (PECVD), involves the use of plasma to activate the ionization and dissociation of hydrocarbon precursors at relatively low temperatures, enabling graphene growth directly on dielectric or semiconducting substrates. PECVD provides several advantages, including reduced synthesis temperatures compared to traditional CVD (>1000 °C), lower energy consumption, and the ability to use a wider range of substrates without risking thermal damage [15,16].

In PECVD systems, remote plasma configurations are typically used to avoid radiation-induced defects or the total etching of the growing graphene film, facilitating the synthesis of high-quality planar graphene layers [17]. Direct plasma systems; however, are more challenging to control due to their higher radiation effects, but they have shown the potential to produce both planar and vertical graphene structures [17,18,19,20]. In our previous research, we demonstrated that it is possible to control the orientation of graphene flakes during direct plasma-based growth on semiconducting and dielectric substrates at temperatures ranging from 700 to 800 °C [17,19,20]. Despite this, there are relatively few studies on graphene synthesis using direct plasma systems [17,18,19,20].

In the present study, we explore the potential for reducing the direct synthesis temperature of graphene through the optimization of hydrogen and methane gas flows and process pressure. These parameters are crucial for balancing the etching and growth processes that govern the quality and properties of the synthesized graphene. By adjusting the flow rates of hydrogen and methane, we investigate how the interplay between these gasses influences the formation of both pristine and hydrogenated graphene at lower temperatures. Our findings demonstrate the feasibility of synthesizing hydrogenated graphene at significantly lower temperatures than conventional pristine graphene, which opens new avenues for low-temperature synthesis techniques and broadens the range of potential substrates that can be used in graphene-based applications.

## 2. Results

**Figure 1:**
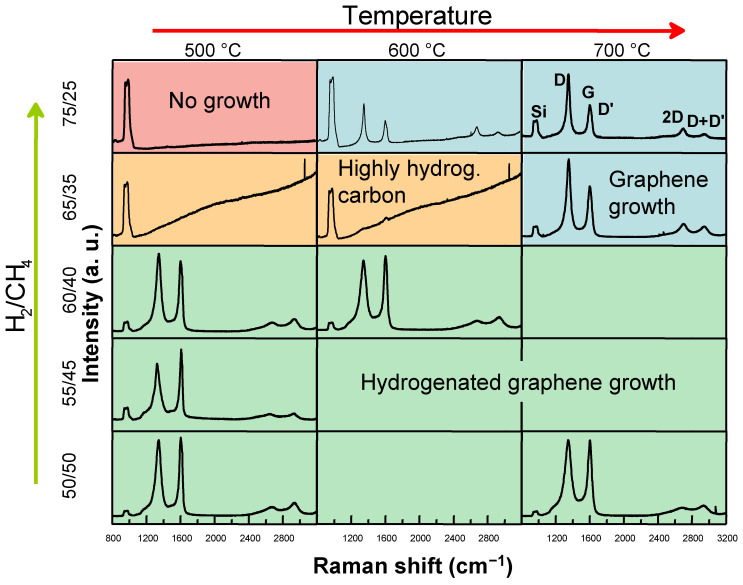
Characteristic Raman spectra of graphene, highly hydrogenated carbon and hydrogenated graphene films grown at different temperatures and H_2_ to CH_4_ gas flow ratios. The total gas flow was 100 sccm and the work pressure was 20 mBar.

In our previous research, the lowest temperature achieved for the direct synthesis of graphene was 700 °C. In this study, we aimed to further reduce the synthesis temperature by optimizing the hydrogen and methane gas flow rates and the process pressure. Initially, experiments were conducted at a working pressure of 20 mBar. Figure 1 illustrates that, at 700 °C, few-layer graphene can be synthesized using gas flow rates of 75 sccm H₂ and 25 sccm CH_4_. The Raman spectra of the synthesized graphene reveal prominent G and 2D peaks [21,22] alongside defect-related D [21,22] and D+D′ peaks [23,24,25,26,27]. Additionally, a shoulder on the G peak indicates the presence of the D′ peak, another defect-associated feature [21,22]. The pronounced D peak is characteristic of graphene directly synthesized on dielectric and semiconducting substrates, and it is attributed to the nanocrystalline structure of the material.

Increasing the methane flow to 35 sccm while reducing the hydrogen flow to 65 sccm still resulted in graphene growth at 700 °C. Further adjustments, such as decreasing the hydrogen flow to 50 sccm and increasing the methane to 50 sccm, significantly reduced the I(D)/I(G) intensity ratio, indicating fewer defects. However, this adjustment also markedly decreased the intensity of the 2D peak, which became less prominent than the D+D′ peak. The D+D′ peak is primarily associated with on-site defects, such as hydrogen atoms covalently bonded to carbon atoms in the graphene lattice. Prior studies [23,24,25,26,27] and our recent work [28] support this observation. Furthermore, the hydrogenation of pristine graphene reduces the 2D band intensity while enhancing the D+D′ band, with an inverse relationship between these bands’ intensities confirming that hydrogenated graphene growth occurred under these conditions.

Lowering the synthesis temperature to 600 °C, with 75 sccm hydrogen and 25 sccm methane flows, still resulted in graphene growth. However, the I(G)/I(Si) ratio was significantly lower than that of the sample grown at 700 °C, indicating a reduced suppression of the substrate’s Raman signal (Table S1). The I(2D)/I(G) ratio increased, suggesting the growth of fewer graphene layers (Table S1). The I(2D)/I(G) ratio values were typical for bilayer graphene [29]. Reducing the hydrogen flow to 65 sccm while increasing the methane flow to 35 sccm led to the emergence of a strong photoluminescence (PL) background in the Raman spectra that is typical of highly hydrogenated amorphous carbon film (~40–50 at.% hydrogen) growth [29]. However, adjusting the gas flow to 60 sccm H₂ and 40 sccm of CH₄ enabled the synthesis of hydrogenated graphene. Similar results were observed at a synthesis temperature of 500 °C. These findings suggest that increasing methane flow and reducing hydrogen flow promote the growth of hydrogenated graphene, which can be achieved at lower temperatures than with “conventional” pristine graphene deposition.

**Figure 2:**
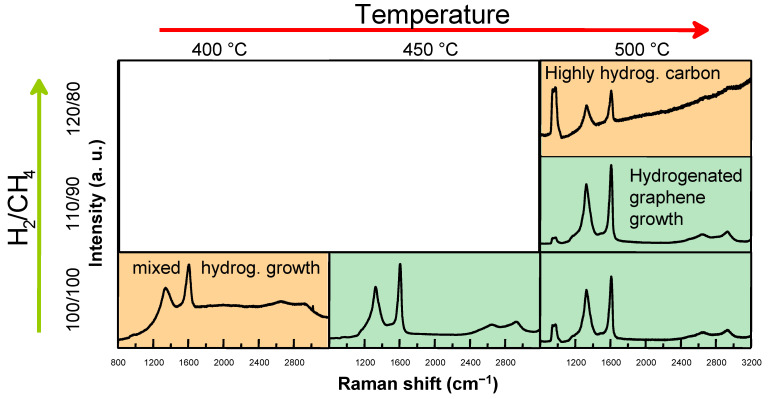
Characteristic Raman spectra of graphene and highly hydrogenated carbon films grown at different temperatures and H_2_ to CH_4_ gas flow ratios. The total gas flow was 200 sccm and the work pressure was 20 mBar.

At 500 °C, a total gas flow of 200 sccm with a 1:1 H_2_:CH_4_ ratio still resulted in hydrogenated graphene growth (Figure 2). However, increasing the hydrogen flow to 120 sccm while decreasing methane to 80 sccm caused the Raman spectra to exhibit a PL background, with the 2D and D+D′ peaks nearly disappearing. Nonetheless, the D and G peaks remained prominent. Notably, a hydrogenated graphene layer was successfully grown at 450 °C with 50 sccm H_2_ and 50 sccm CH_4_ flows. At 400 °C, the Raman spectra featured characteristics of hydrogenated graphene, including 2D and D+D′ peaks, along with a broad PL lump. These observations suggest that the sample synthesized at 400 °C was transitioning from hydrogenated graphene to a highly hydrogenated carbon film. However, in this case, the Raman spectra luminescence feature differs from other highly hydrogenated carbon films observed in this study. The presence of defective polycyclic aromatic hydrocarbon bonds is plausible [30].

**Figure 3:**
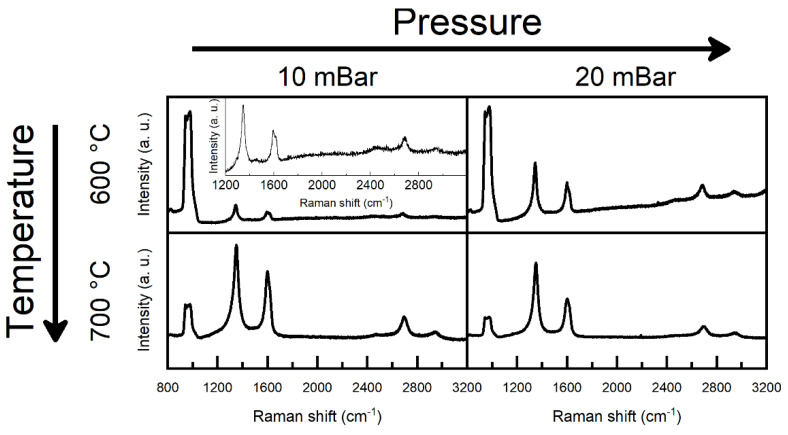
**The** Raman spectra of graphene grown under different synthesis pressures and temperatures. The hydrogen gas flow was 75 sccm and the methane gas flow was 25 sccm.

**Figure 4:**
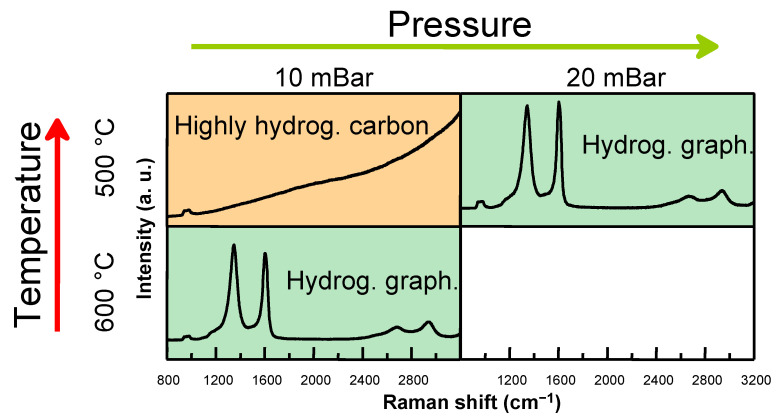
Raman spectra of hydrogenated graphene and highly hydrogenated carbon films grown under different temperature and synthesis pressure. The hydrogen gas flow was 50 sccm and the methane gas flow was 50 sccm.

Reducing the synthesis pressure from 20 mBar to 10 mBar maintained graphene growth at 700 and 600 °C temperatures under 75 sccm H_2_ and 25 sccm CH_4_ flows (Figure 3). The I(G)/I(Si) ratio was substantially lower, and the I(2D)/I(G) ratio was higher for graphene grown at 600 °C and 10 mBar pressure than for the 20 mBar sample, implying a slower graphene growth process (Table S1). In addition, at 500 °C, hydrogenated graphene did not form if 10 mBar work pressure was used (Figure 4). Instead, the Raman spectra displayed a PL background and a substrate-related peak. Under these conditions, hydrogenated graphene synthesis was observed only at 600 °C (Figure 4).

At 500 °C and 10 mBar pressure, varying the total gas flow revealed further insights (Figure S1). Decreasing the total flow to 100 sccm resulted in highly hydrogenated carbon formation, whereas increasing the total flow to 200 sccm led to D and G peaks appearing in the Raman spectra, with a concomitant reduction being seen in the PL background (Figure 4). While using 20 mBar pressure at 500 °C temperature, in all cases, hydrogenated graphene was grown irrespectively of the total gas flow used (Figure S1).

**Figure 5:**
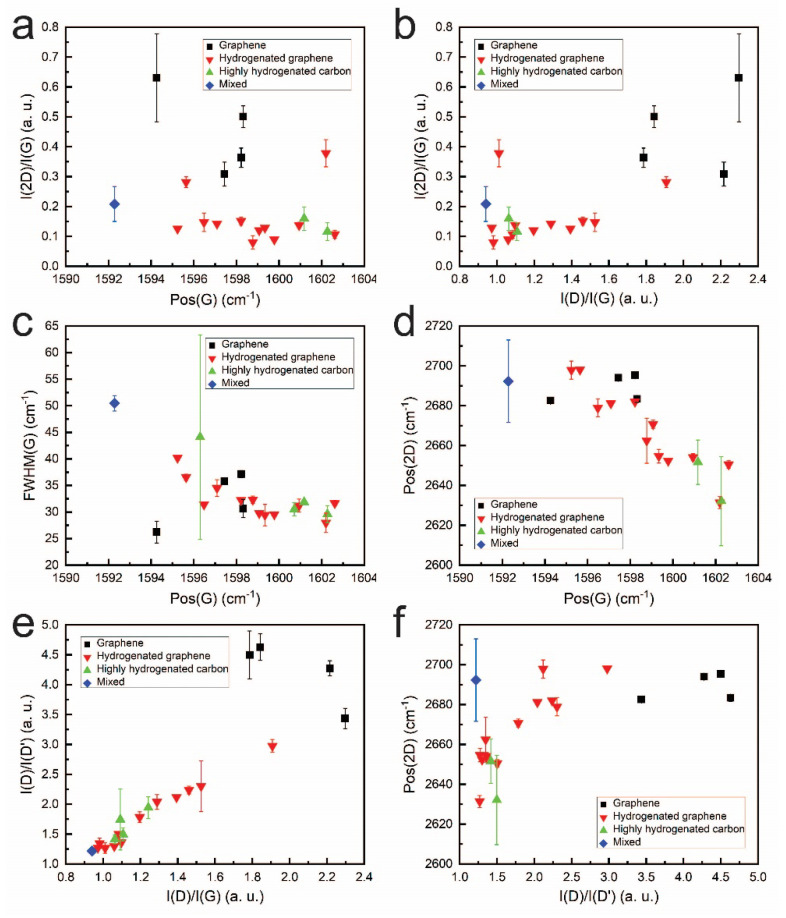
Plots of I(2D)/I(G) vs. Pos(G) (**a**); I(2D)/I(G) vs. I(D)/I(G) (**b**); FWHM(G) vs. Pos(G) (**c**); Pos(2D) vs. Pos(G) (**d**); I(D)/I(D’) vs. I(D)/I(G) (**e**); Pos(2D) vs. I(D)/I(D’) (**f**).

The Raman scattering spectra parameters of the samples were further analyzed to investigate their structural characteristics. The I(2D)/I(G) ratio is commonly employed as an indicator of the number of graphene layers [31]. However, this ratio can be influenced by graphene doping and the presence of defects, both of which can reduce the I(2D)/I(G) ratio. Specifically, doping can result in a reduced I(2D)/I(G) ratio accompanied by an upshift in the Pos(G) position [32,33]. In contrast, our results exhibit different behavior (Figure 5a). Therefore, the changes in this study in the I(2D)/I(G) ratio are not attributed to the possible graphene doping.

While an increase in defect density is typically associated with a decrease in the I(2D)/I(G) ratio [34,35], the hydrogenated graphene samples in this study exhibited an increase in the I(2D)/I(G) ratio with an increasing I(D)/I(G) ratio (Figure 5b). Consequently, the I(2D)/I(G) ratio was utilized to estimate the number of graphene layers. Notably, for most hydrogenated graphene samples, the I(2D)/I(G) ratio was significantly lower than that observed for pristine graphene (Figure S1), indicative of a greater number of layers.

The full width at half maximum of the G peak (FWHM(G)) decreased as Pos(G) shifted upward (Figure 5c), consistent with graphene doping effects [32,33]. Furthermore, Pos(2D) exhibited a downward shift in response to the Pos(G) upshift (Figure 5d), a behavior characteristic of n-type graphene doping [32,36]. For most hydrogenated graphene samples, Pos(2D) was less red-shifted than for conventional pristine graphene, indicating a more pronounced doping effect in hydrogenated graphene [37,38].

Consistent with our previous studies, substrate-induced graphene self-doping can be presumed [17,19,39,40]. Numerous research papers have documented this mechanism, highlighting the critical role of substrate interactions in graphene’s electronic properties [41,42,43,44,45,46,47]. The type of self-doping (p-type or n-type) depends on the substrate material. For instance, studies have demonstrated that the surface orientation of SiO_2_ significantly influences graphene’s doping characteristics. An O-polar SiO_2_ surface with dangling bonds induces p-type doping in graphene [43], whereas a Si-polar surface with dangling bonds results in n-type self-doping [43]. These findings suggest that the direct graphene synthesis process intrinsically modifies the substrate surface or interface composition, facilitating the transfer of induced charges to the graphene layer.

The I(D)/I(D′) ratio was observed to increase with the I(D)/I(G) ratio (Figure 5e). For some samples, the I(D)/I(D′) ratio was consistent with boundary defects (≈3.5). However, in most cases, the ratio suggested the co-dominance of on-site defects and boundary defects or the exclusive presence of on-site defects [48]. On-site defects in graphene are commonly attributed to adsorbed hydrogen atoms [49], corroborating the Raman scattering spectra and confirming the growth of hydrogenated graphene. Some correlation was observed between Pos(2D) and the I(D)/I(D′) ratio for hydrogenated graphene samples, indicating the graphene hydrogenation influence on graphene doping (Figure 5f). It should be noted that n-type doping of the graphene due to the hydrogenation was already reported in [50,51].

The I(G)/I(Si) peak ratio was evaluated as an additional measure of film thickness [19,31]. No dependence of the I(2D)/I(G) ratio on the I(G)/I(Si) ratio was observed for most samples (Figure S2), suggesting that I(G)/I(Si) is not directly related to the graphene layer number in hydrogenated graphene films. However, for hydrogenated graphene samples, the I(G)/I(Si) ratio was significantly smaller compared to pristine graphene, indicating the formation of thicker or less heterogeneous films.

The effects of synthesis conditions on the Raman scattering spectra parameters revealed that I(2D)/I(G) was more dependent on the graphene type (pristine or hydrogenated) than on deposition conditions for samples grown using a total gas flow of 100 sccm (Figures S3–S5). A decrease in synthesis temperature corresponded to a reduction in the I(D)/I(D′) ratio (Figures S3 and S5). Additionally, reducing the H_2_/CH_4_ gas flow ratio at 700 °C led to a decrease in the I(D)/I(D′) ratio and an increase in the I(G)/I(Si) ratio (Figure S3). Lowering the synthesis temperature from 700 °C to 600 °C or 500 °C enhanced graphene self-doping effects (Figure S3). However, a further temperature reduction to 400 °C diminished the self-doping effect, probably due to the changes in the nature of C-H bonds (Figure S5). It seems that decreasing the work pressure to 10 mbar promoted graphene self-doping (Figure S6).

**Figure 6:**
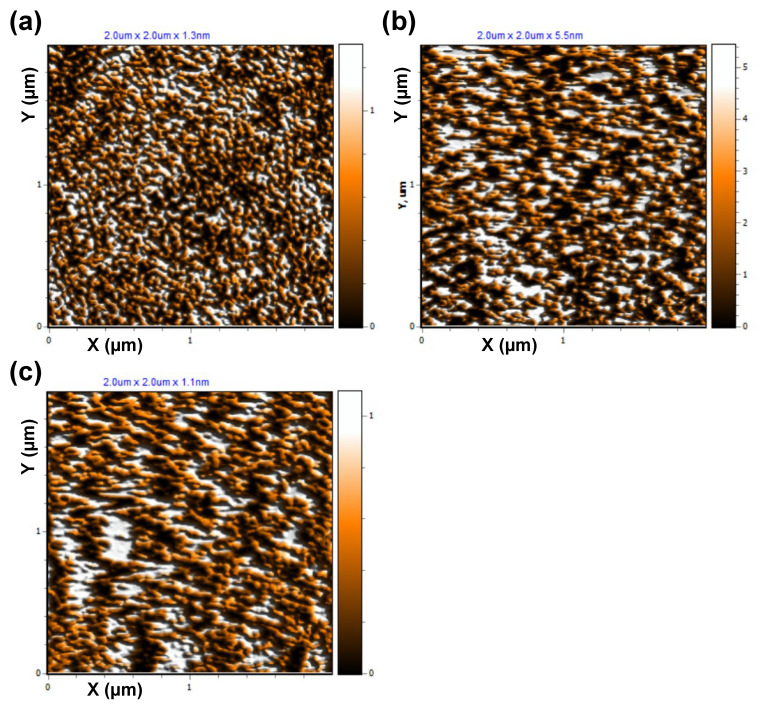
(AFM) topographical images of (**a**) pristine graphene, (**b**) hydrogenated graphene prepared at 50 sccm CH_4_ and 50 sccm H_2_ gas flow rates, and (**c**) hydrogenated graphene prepared at 100 sccm CH_4_ and 100 sccm H₂ gas flow rates.

The atomic force microscopy (AFM) topographical images depicted in Figure 6 provide detailed insights into the surface morphologies of pristine graphene and hydrogenated graphene samples synthesized via plasma-enhanced chemical vapor deposition (PECVD). The pristine graphene, shown in Figure 6a, was synthesized at a substrate temperature of 700 °C utilizing gas flow rates of 75 sccm CH_4_ and 25 sccm H_2_. In contrast, the hydrogenated graphene samples were fabricated at a lower substrate temperature of 500 °C, employing a 1:1 gas flow ratio. Specifically, the flow rates used were 50 sccm CH_4_ and 50 sccm H_2_ (Figure 6b) and 100 sccm CH_4_ and 100 sccm H_2_ (Figure 6c).

The AFM image of pristine graphene (Figure 6a) shows a relatively smooth surface characterized by a granular texture, indicative of a continuous and uniform graphene layer. A quantitative surface morphology analysis reveals an average height (*Z_mean_*) of 0.68 nm with a root mean square roughness (*R_q_*) of 0.37 nm. These values are consistent with those reported in earlier studies on PECVD-grown graphene [19], suggesting that the graphene layer is largely free of significant defects or discontinuities. The surface skewness (*R_sk_*) of −0.06 indicates a symmetrical height distribution with slight depressions. At the same time, the kurtosis (*R_ku_*) value of 1.98 implies that the surface morphology has no extreme peaks or valleys dominating the surface features.

Conversely, the hydrogenated graphene samples (Figure 6b) prepared at 50 sccm CH_4_ and 50 sccm H_2_ gas flow rates demonstrate a markedly rougher surface morphology, reflecting significant structural changes induced by incorporating hydrogen atoms into the graphene lattice. The average height of the surface increases to 2.81 nm, with a corresponding rise in the *R_q_* to 1.69 nm. This increase in roughness indicates surface distortions and the introduction of structural irregularities due to hydrogenation. The *R_sk_* of −0.05 indicates a symmetrical distribution of surface heights similar to that of pristine graphene, but the *R_ku_* value of 1.89 suggests a slightly broader distribution of height features. These changes are in line with Raman observations which suggest that the hydrogenation process disrupts the sp^2^-hybridized carbon network, replacing it with localized sp^3^ bonds, which lead to out-of-plane buckling and increased nanoscale surface roughness with larger, irregularly shaped grains or clusters.

The hydrogenated graphene sample (Figure 6c), prepared at 100 sccm CH₄ and 100 sccm H_2_ gas flow rates, exhibits a surface morphology between pristine (Figure 6a) and hydrogenated graphene (shown in Figure 6b). A quantitative analysis reveals a *Z_mean_* of 0.57 nm and an *R_q_* of 0.30 nm. These values suggest a surface slightly smoother than pristine graphene but with limited roughness. The *R_sk_* of 0.01 indicates a nearly symmetrical height distribution, while the kurtosis *R_ku_* value of 2.04 reflects a slightly sharper distribution of peaks and valleys. The surface morphology suggests smaller, more uniform grains compared to the hydrogenated graphene shown in Figure 6b.

The morphological differences between the pristine graphene and hydrogenated graphene can be attributed to the distinct synthesis conditions and the effect of hydrogen incorporation during PECVD. The pristine graphene synthesized at a higher temperature and with reduced hydrogen flow retains a planar sp^2^-hybridized structure, resulting in a smoother and more uniform surface. In contrast, the hydrogenation process carried out at a lower temperature introduces localized distortions in the graphene lattice as sp^3^-hybridized carbon-hydrogen bonds. These distortions disrupt the continuity of the graphene layer, leading to increased surface heterogeneity with the formation of larger grains or clusters. However, by adjusting the synthesis parameters, it is possible to tune the surface roughness to levels close to that of pristine graphene or to achieve even smoother surfaces.

These observations highlight the role of PECVD parameters in tailoring the surface morphology and structural properties of graphene. The smoother topography is suitable for electronic and optoelectronic applications requiring uniform layers, while the enhanced surface roughness can be advantageous for applications such as catalysis, sensing, or energy storage, where increased surface areas and reactive sites are desirable.

**Figure 7:**
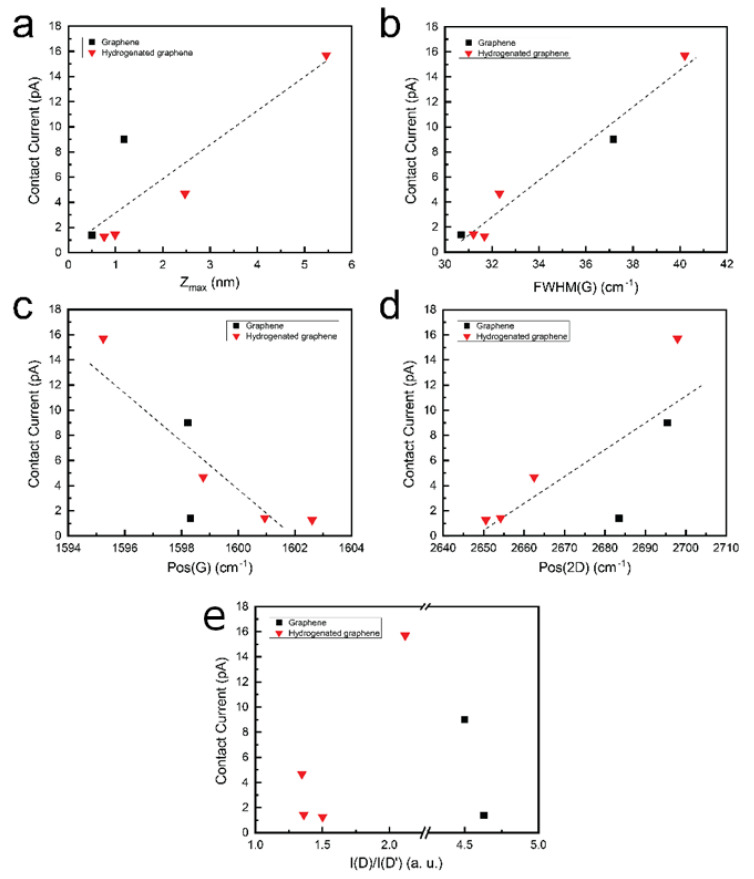
Mean Z(pA) values plotted against (**a**) Zmax, (**b**) FWHM(G), (**c**) Pos(G), (**d**) Pos(2D) and (**e**) I(D)/I(D’). Dashed gray lines indicate trends in the graphs.

The contact current of the graphene and hydrogenated graphene samples was evaluated using Conductive Atomic Force Microscopy (CP-AFM), with the mean contact current values being plotted against various parameters, as illustrated in Figure 7. The results indicate that the contact current of hydrogenated graphene samples increased with surface roughness. The contact current of hydrogenated graphene was also found to correlate with specific Raman spectroscopy features, including a broadening of the G peak, a shift in the G peak to higher wavenumbers (Pos(G)), and a downshift in the 2D peak (Pos(2D)). This trend was consistent with the behavior observed in pristine graphene samples. Notably, the pristine graphene synthesis temperature reduction from 700 °C to 600 °C resulted in significant contact current reduction—from 9 to the 1.39 pA (Table S2). The tendencies revealed in Figure 6 mean that the reduction in contact current for both graphene and hydrogenated graphene can be attributed to self-doping phenomena. Thus, the self-doping phenomena should be suppressed to increase the contact current. Furthermore, no dependence of contact current on the I(D)/I(D’) ratio was found for either hydrogenated graphene or pristine graphene, suggesting that the relationship between graphene’s electrical properties, self-doping, and the type of defects, as well as hydrogenation, is not straightforward. Some competition between the substrate-induced graphene self-doping and hydrogenation-induced doping can be presumed. Interestingly, the contact current of hydrogenated graphene can exceed that of pristine graphene despite hydrogenation and the dominance of on-site defects. It indicates that hydrogenation can enhance the contact current of graphene under certain conditions that are independent of the defect type and concentration.

Summarizing, a decrease in graphene synthesis temperature and a reduction in the hydrogen/methane gas flow ratio facilitated the growth of hydrogenated graphene. Lower synthesis temperatures also enhanced the degree of graphene hydrogenation, enabling the successful synthesis of hydrogenated graphene at temperatures as low as 400–450 °C. Increasing the work pressure proved beneficial in regard t lowering the synthesis temperature required for graphene formation.

The growth of hydrogenated graphene is likely attributed to the incomplete dissociation of methane molecules under these synthesis conditions. Self-doping effects were observed in the synthesized graphene samples. In the hydrogenated graphene, these effects were related to graphene hydrogenation, and the prevailing of the hydrogen-related on-site defects promoted n-type self-doping effects. The degree of self-doping exhibited a non-monotonic dependence on synthesis temperature, while reduced work pressure promoted self-doping.

The observed results can be explained by competition between several processes. These are methane and hydrogen molecules’ dissociation and ionization, graphene/carbon film growth, and carbon etching by hydrogen. Particularly, an increase in the CH_4_ gas flow and in the total gas flow results in decreased power density per methane molecule and less time for methane dissociation [52]. Decreased temperature results in suppressed molecule dissociation [53], an increased hydrogen etching rate [54], and a reduced carbon desorption rate [55]. Decreased pressure increases power density per molecule, enhancing the dissociation [56]. Lower pressure also helps to achieve higher electron mean free paths and higher electron temperatures, subsequently increasing the gas ionization rate and ion bombardment [57].

One of the challenges associated with PECVD-based graphene synthesis is achieving precise control over the number of graphene layers. While our process yielded predominantly bilayer and few-layer graphene, as evidenced by Raman spectroscopy, the scalability of the method may be influenced by factors such as substrate uniformity, plasma distribution, and gas flow dynamics. Variations in these parameters can result in localized inconsistencies in layer thickness, particularly over larger substrate areas.

Additionally, the use of a steel enclosure to shield the substrate from direct plasma exposure, while effective in minimizing excessive etching and providing a possibility to control the orientation of the graphene flakes, may inadvertently create non-uniform deposition conditions at the edges of the sample. This limitation could affect the reproducibility of the process when scaling to larger substrates.

To address these issues, further optimization of the reactor design, including uniform plasma distribution and precise substrate positioning, is necessary.

## 3. Materials and Methods

The graphene was synthesized directly on a 300 nm-thick silicon dioxide (SiO_2_) layer thermally grown on a Si(100) substrate using a gas mixture of hydrogen (H_2_) and methane (CH_4_). The synthesis process was conducted in a Cyrannus microwave plasma-enhanced chemical vapor deposition (PECVD) system (Innovative Plasma Systems (Iplas) GmbH, Troisdorf, Germany). The substrate dimensions were 1 × 1 cm.

Prior to graphene growth, the substrate underwent a pretreatment stage involving hydrogen plasma exposure without any prior wet-chemical treatment. During this step, the plasma power was set at 1.0 kW, the process temperature was maintained at 700 °C, the hydrogen flow rate was 200 sccm, the chamber pressure was 10 mBar, and the treatment duration was 10 min.

Following the pretreatment, CH_4_ gas was introduced into the reaction chamber for graphene deposition. A specially designed enclosure, fabricated from a rectangular non-magnetic steel sheet bent perpendicularly at two points, was utilized to shield the sample from direct plasma interactions. This arrangement minimized excessive etching of the growing graphene layer, ensuring controlled deposition and improved material quality. Synthesis conditions are presented in Table 1.

During graphene synthesis, the chamber pressure was monitored and regulated using an automated feedback system connected to the gas flow controller. A calibrated pressure gauge was employed for real-time measurement and precise control of the chamber pressure throughout the process. Th substrate temperature was controlled using a resistive heater integrated into the PECVD system. The heater’s temperature was monitored by a thermocouple positioned directly beneath the substrate, ensuring accurate temperature measurement.

The Raman scattering spectra of the synthesized samples were recorded using a Renishaw inVia Raman spectrometer (Renishaw, Wotton-under-Edge, UK). A 532 nm wavelength laser was used for excitation with a power of 3 mW. The D, G, D’, and 2D peaks were fitted using the Lorentzian function. The intensity ratio of the 2D to G peaks (I(2D)/I(G)) was used to estimate the number of graphene layers [31]. The intensity ratio of the D to G peaks (I(D)/I(G)) was calculated to assess the defect density of graphene [35,58]. The intensity ratio of the D and D’ peaks was analyzed to determine the dominant defect type [48,49]. The full width at half maximum (FWHM) of the G peak (FWHM(G)) [37,38,49], as well as the positions of the G and 2D peaks (Pos(G) and Pos(2D)) [32,36], were also considered as parameters sensitive to graphene doping and stress. The broad substrate-related peak at ~900 cm^−1^ (associated with SiO_2_/Si) was taken into account, and the I(G)/I(Si) ratio was calculated to further estimate the thickness of the deposited film [19,31,59]. Notably, the I(G)/I(Si) ratio increases with the number of graphene layers in planar graphene [31,59] and with the total film thickness for vertical graphene and other non-planar graphene structures [19]. In this study, the substrate-related peak at ~900 cm^−1^ was used for analysis in place of the main silicon-related peak at ~520 cm^−1^ to avoid issues related to peak intensity saturation.

The surface morphology and contact current of the samples were investigated using atomic force microscopy (AFM, NanoWizard^®^3, JPK Instruments, Bruker Nano GmbH, Berlin, Germany). Morphological images were acquired with an ACTA probe (Applied NanoStructures, Inc., Mountain View, CA, USA) operating in tapping mode with a tip radius of curvature of 6 nm. The contact current, which serves as a measure of the samples’ electrical conductivity, was examined using contact-mode conductive atomic force microscopy (C-AFM) with a Pt/Ir-coated ANSCM-PT silicon tip probe (thickness: 25 ± 5 nm; App-Nano, Mountain View, CA, USA). Both the reflex and tip sides of the probe were coated. The spring constant of the ANSCM probe was 1.6 N/m with a tetrahedral tip shape, a radius of curvature (ROC) of 30 nm, a height of 14–16 μm, and a frequency of 61 kHz. Special Ag electrodes were fabricated on the graphene layer to investigate the surface contact current. The electrical current was measured as a function of the applied bias voltage (−10 to 10 mV). All measurements were performed at room temperature in ambient air, with a bit noise of 55 fA at a current gain of 1x.

## 4. Conclusions

This study successfully demonstrates the potential for directly synthesizing hydrogenated graphene at significantly reduced temperatures using plasma-enhanced chemical vapor deposition (PECVD). The optimization of gas flow rates, pressure, and temperature enabled the controlled synthesis of both pristine and hydrogenated graphene, with distinct structural and electronic properties.

Few-layer graphene can be synthesized at 700 °C, and bilayer graphene can be grown at a 600 °C temperature using specific gas flow ratios (e.g., 75 sccm H_2_, 25 sccm CH_4_). However, the contact current of the few-layer graphene estimated by CP-AFM was much larger than that of the bilayer graphene. Increasing methane flow while reducing hydrogen flow promotes hydrogenation and decreases defect density, as evidenced by changes in Raman spectral features, including the I(D)/I(G) and I(2D)/I(G) ratios. A 1:1 H_2_:CH_4_ ratio enables the synthesis of hydrogenated graphene at temperatures as low as 450 °C, suggesting the feasibility of low-temperature processes for specific graphene-based applications. At 400 °C, the material transitions to the mixture of the hydrogenated graphene and a version of the highly hydrogenated carbon, marked by significant photoluminescence (PL) backgrounds in Raman spectra. These transitions can be explained by the formation of defective polycyclic aromatic hydrocarbon bonds, particularly at extreme hydrogenation levels.

The Raman analysis indicates the co-dominance of on-site and boundary defects in hydrogenated graphene, with the presence of D and D+D′ peaks correlating to localized sp^3^ hybridization due to adsorbed hydrogen atoms.

It seems that graphene hydrogenation promoted graphene n-type doping. Lower synthesis temperatures (500–600 °C) enhance self-doping effects. However, growth at 400 °C temperature results in reduced doping, probably due to the changes in the character of the C-H bonds.

Atomic force microscopy (AFM) revealed distinct surface morphologies for pristine and hydrogenated graphene. Adjusting the gas flow and temperature allowed for control over surface morphology, enabling the synthesis of graphene and hydrogenated graphene films with tailored roughness and grain sizes.

CP-AFM measurements indicate that hydrogenated graphene can exhibit enhanced electrical performance compared to pristine graphene under specific conditions. This finding highlights the potential utility of hydrogenation for tuning graphene’s electronic properties.

Our findings illustrate that precise control over PECVD synthesis parameters enables the growth of graphene with desired properties, paving the way for low-temperature, scalable fabrication of functional graphene materials. The demonstrated methodologies hold promise in terms of advancing graphene integration into next-generation technologies.

## Figures and Tables

**Table 1:** Technological process synthesis conditions.

Sample No	Temperature (°C)	H_2_ Flow (sccm)	CH_4_ Flow (sccm)	Pressure (mBar)
1	700	75	25	20
2	700	75	25	10
3	600	75	25	20
4	600	75	25	10
5	500	75	25	20
6	700	65	35	20
7	600	65	35	20
8	500	65	35	20
9	600	60	40	20
10	500	60	40	20
11	500	55	45	20
12	700	50	50	20
13	600	50	50	10
14	500	50	50	20
15	500	50	50	10
16	500	120	80	20
17	500	110	90	20
18	500	100	100	20
19	450	100	100	20
20	400	100	100	20
21	500	100	100	10
22	500	75	75	20
23	500	80	70	20

## Data Availability

Data are contained within the article.

## Supplementary Materials

The following supporting information can be downloaded at: https://www.mdpi.com/article/10.3390/molecules30010033/s1. Table S1: Samples‘ Raman scattering spectra parameters. Table S2: Selected samples‘ average AFM feature height (Z(nm)) and contact current. Figure S1: Raman spectra of the samples grown using different total gas flow and synthesis pressure. Figure S2: Plot I(2D)/I(G) vs. I(G)/I(Si). Figure S3: H_2_ and CH_4_ gas flow ratios and temperature effects on samples‘ Raman scattering spectra parameters. The work pressure was 20 mBar and the total gas flow was 100 sccm. Figure S4: Hydrogen and methane gas flow ratio effects on samples‘ Raman scattering spectra parameters. The work pressure was 20 mBar, the synthesis temperature was 500 °C, and the total gas flow was 200 sccm. Figure S5: Temperature effects on samples‘ Raman scattering spectra parameters. The work pressure was 20 mBar, the hydrogen gas flow was 100 sccm, and the methane gas flow was 100 sccm. Figure S6: Total gas flow and work pressure effects on samples‘ Raman scattering spectra parameters. The synthesis temperature was 500 °C and the hydrogen and methane gas flow ratio was equal to 1.
