# Supplementary material for: Advancing Graphene Synthesis: Low-Temperature Growth and Hydrogenation Mechanisms Using Plasma-Enhanced Chemical Vapor Deposition
Source: Molecules. 2024 Dec 25;30(1):33. doi: 10.3390/molecules30010033 (PMC11720853; doi:10.3390/molecules30010033)
Supplement: Supplementary file 1 [file molecules-30-00033-s001.zip › molecules-3382842-supplementary.pdf]

Table S1. Samples' Raman scattering spectra parameters.

Sample No	T (°C)	H2 flow (sccm)	CH4 flow (sccm)	P (mBar)	I(2D)/I(G)	I(D)/I(G)	FWHM(G) (cm^{-1})	Pos(G) (cm^{-1})	Pos(2D) (cm^{-1})	I(D)/I(D')	I(G)/I(Si)
1	700	75	25	20	0.31	2.22	35.8	1597.4	2694.1	4.28	1.77
2	700	75	25	10	0.3613	1.79	37.2	1598.2	2695.4	4.50	1.6073
3	600	75	25	20	0.50	1.84	30.7	1598.3	2683.4	4.63	0.24
4	600	75	25	10	0.63	2.30	26.2	1594.3	2682.6	3.44	0.04
5	500	75	25	20							
6	700	65	35	20	0.28	1.91	36.5	1595.6	2698.2	2.98	3.82
7	600	65	35	20		1.24	30.5	1600.7		1.944	0.04
8	500	65	35	20							
9	600	60	40	20	0.14	1.29	34.5	1597.1	2681.3	2.04	7.22
10	500	60	40	20	0.15	1.53	31.4	1596.5	2679	2.30	6.35
11	500	55	45	20	0.11	1.08	31.7	1602.6	2650.6	1.50	2.91
12	700	50	50	20	0.13	1.39	40.2	1595.2	2697.9	2.12	11.86
13	600	50	50	10	0.15	1.46	32.3	1598.2	2682	2.24	18.71
14	500	50	50	20	0.12	1.20	29.8	1599.1	2670.8	1.79	12.18
15	500	50	50	10		1.09	44.1	1596.3		1.74	0.13
16	500	120	80	20	0.16	1.06	31.9	1601.2	2651.7	1.42	0.47
17	500	110	90	20	0.38	1.01	28	1602.2	2631.4	1.27	0.13
18	500	100	100	20	0.14	1.1	31.2	1600.9	2654.2	1.36	5.93
19	450	100	100	20	0.13	0.97	29.4	1599.3	2654.7	1.271	1.80
20	400	100	100	20	0.21	0.94	50.5	1592.3	2692.3	1.22	23.86
21	500	100	100	10	0.12	1.11	29.6	1602.3	2632.2	1.50	0.34
22	500	75	75	20	0.08	0.98	32.3	1598.8	2662.5	1.35	38.63
23	500	80	70	20	0.09	1.06	29.6	1599.8	2652.4	1.30	19.32

Table S2. Selected samples' average AFM feature height (Z(nm)) and contact current

Sample No	Temperature (°C)	H_2 flow (sccm)	CH_4 flow (sccm)	Pressure (mBar)	Z (nm)	Contact current (pA)
2	700	75	25	10	1.18	9
3	600	75	25	20	0.499	1.39
11	500	55	45	20	0.76	1.27
12	700	50	50	20	5.46	15.7
18	500	100	100	20	0.99	1.43
22	500	75	75	20	2.47	4.68

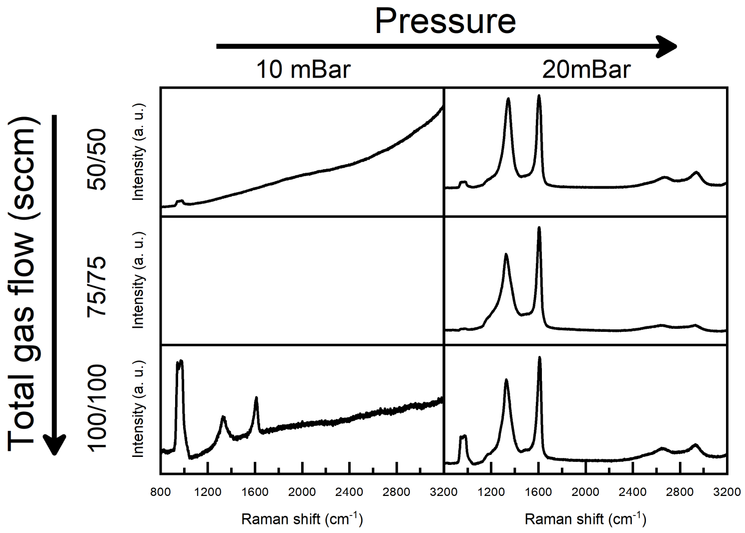

Figure S1. Raman spectra of the samples grown using different total gas flow and synthesis pressure.

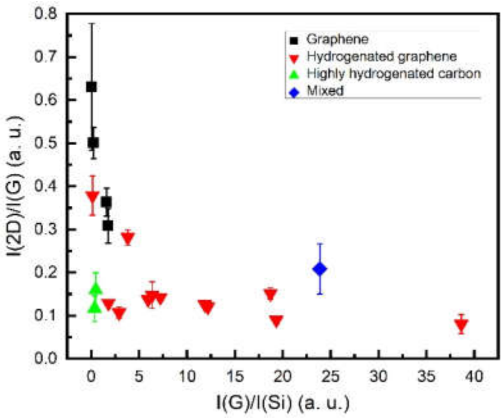

Figure S2. Plot I(2D)/I(G) Vs I(G)/I(Si)

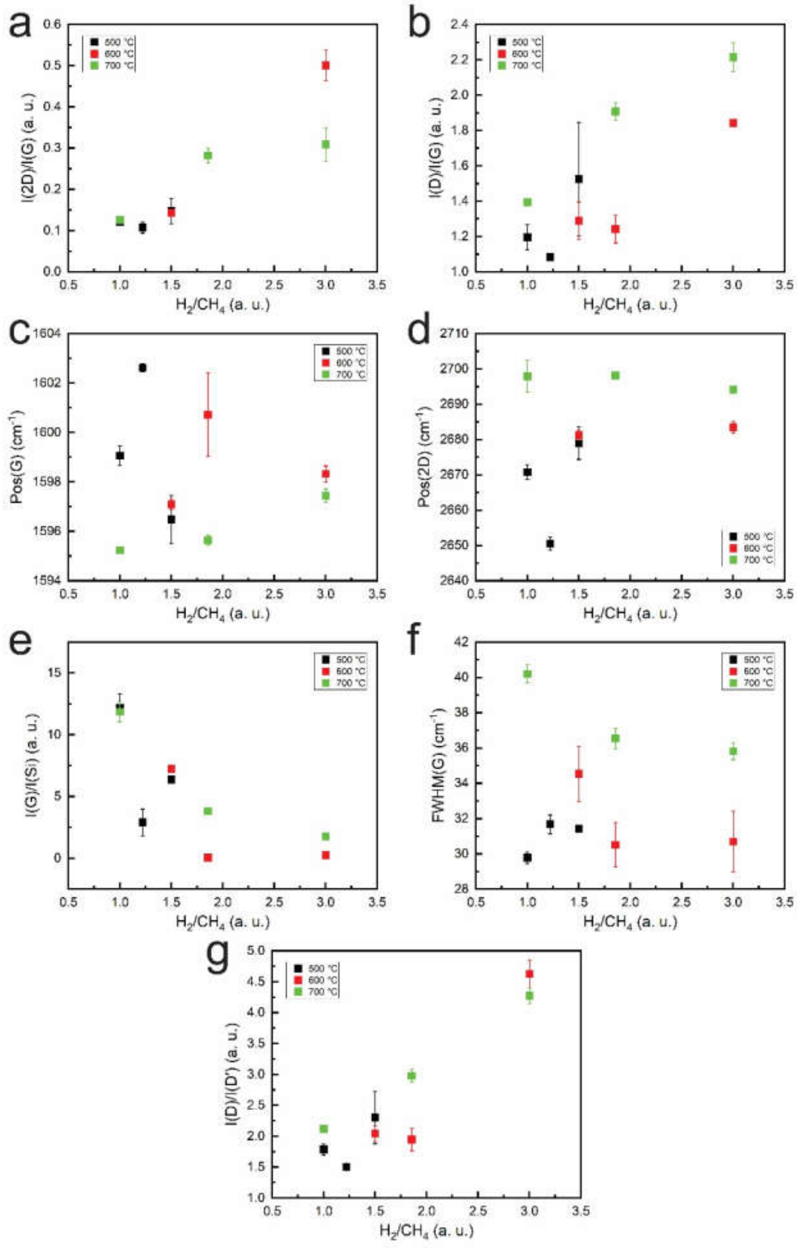

Figure S3. H_2 and CH_4 gas flow ratios and temperature effects on samples' Raman scattering spectra parameters. The work pressure was 20 mBar and total gas flow 100 sccm.

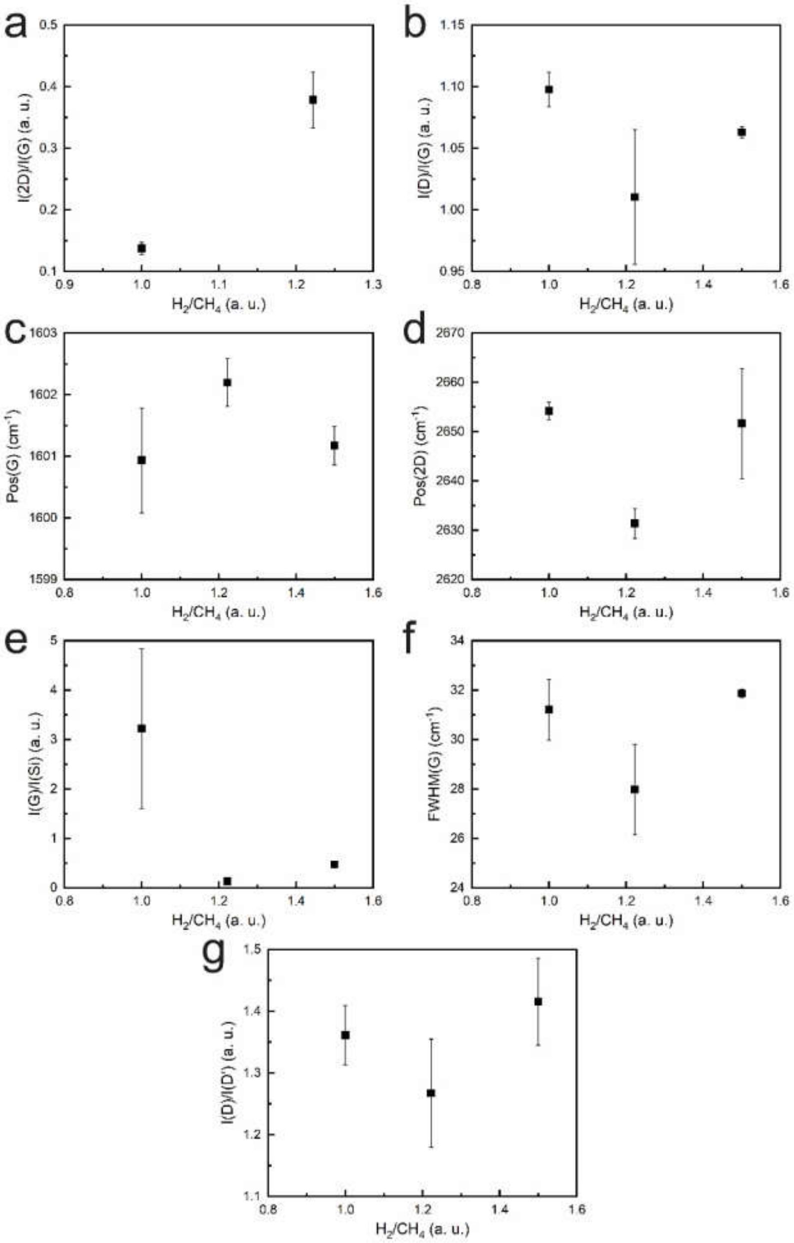

Figure S4. Hydrogen and methane gas flows ratio effects on samples' Raman scattering spectra parameters. The work pressure was 20 mBar, synthesis temperature was 500 ^{o}C, and total gas flow was 200 sccm.

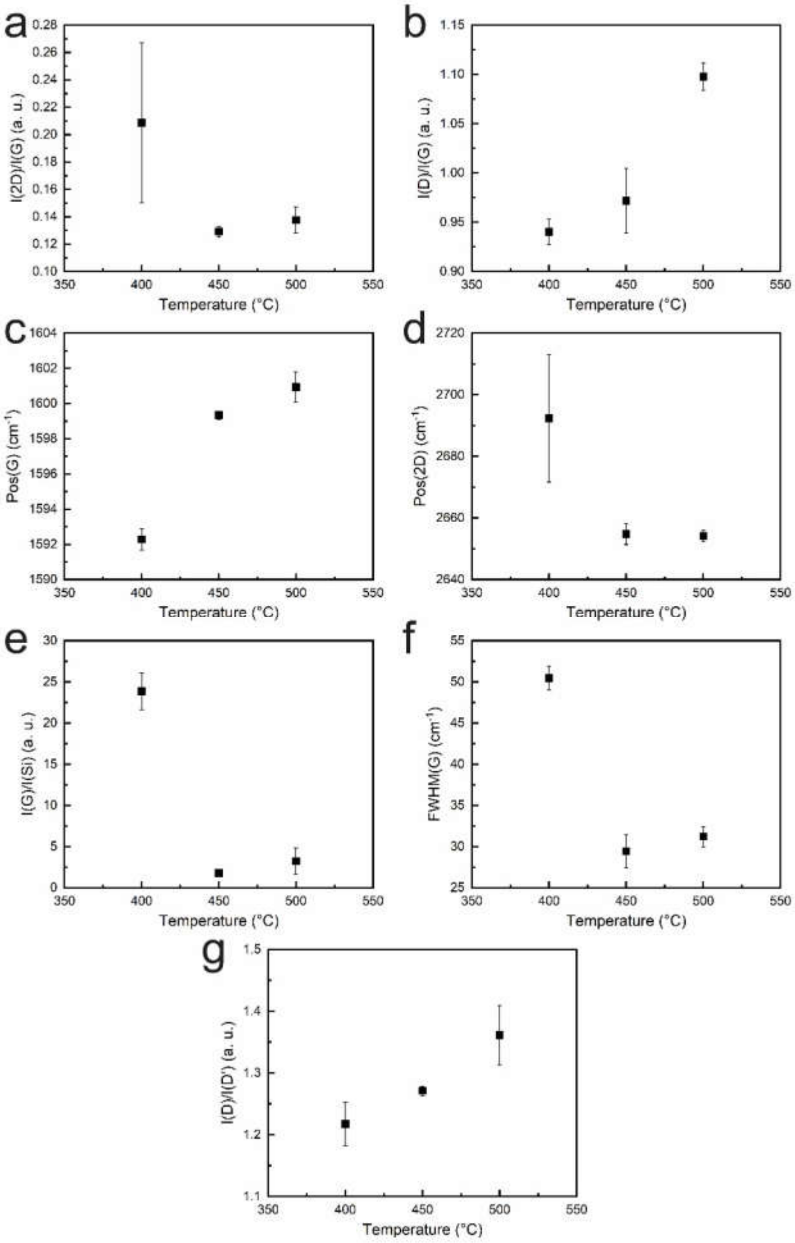

Figure S5. Temperature effects on samples' Raman scattering spectra parameters. The work pressure was 20 mBar, hydrogen gas flow was 100 sccm and methane gas flow was 100 sccm.

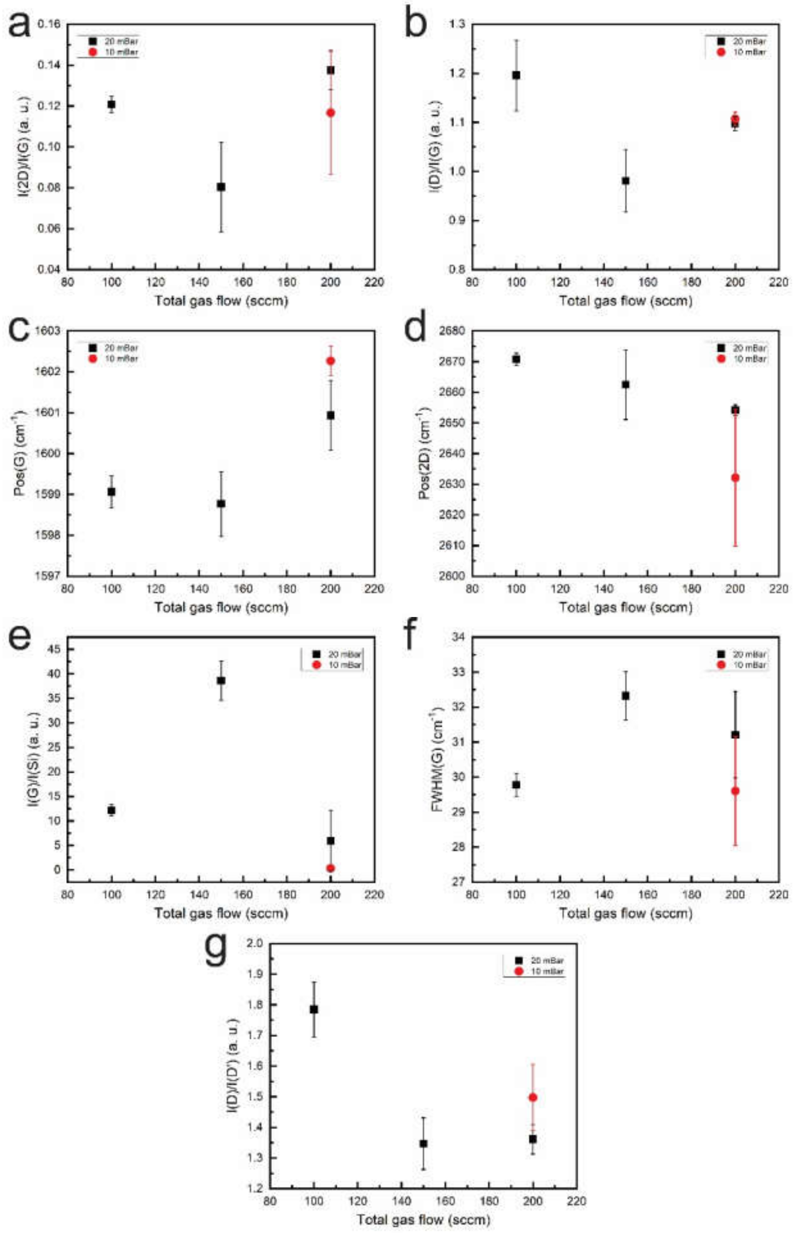

Figure S6. Total gas flow and work pressure effects on samples' Raman scattering spectra parameters. The synthesis temperature was 500 °C, hydrogen and methane gas flows ratio was equal to 1.
